# Prediction of repurposed drugs for Coronaviruses using artificial intelligence and machine learning

**DOI:** 10.1016/j.csbj.2021.05.037

**Published:** 2021-05-24

**Authors:** Akanksha Rajput, Anamika Thakur, Adhip Mukhopadhyay, Sakshi Kamboj, Amber Rastogi, Sakshi Gautam, Harvinder Jassal, Manoj Kumar

**Affiliations:** a Virology Unit and Bioinformatics Centre, Institute of Microbial Technology, Council of Scientific and Industrial Research (CSIR), Sector 39-A, Chandigarh 160036, India; b Academy of Scientific and Innovative Research (AcSIR), Ghaziabad 201002, India

**Keywords:** Coronaviruses, COVID-19, SARS-CoV-2, Drug repurposing, Machine learning, AI, Chemical descriptors

## Abstract

The world is facing the COVID-19 pandemic caused by Severe Acute Respiratory Syndrome Coronavirus 2 (SARS-CoV-2). Likewise, other viruses of the *Coronaviridae* family were responsible for causing epidemics earlier. To tackle these viruses, there is a lack of approved antiviral drugs. Therefore, we have developed robust computational methods to predict the repurposed drugs using machine learning techniques namely Support Vector Machine, Random Forest, *k*-Nearest Neighbour, Artificial Neural Network, and Deep Learning. We used the experimentally validated drugs/chemicals with anticorona activity (IC_50_/EC_50_) from ‘DrugRepV’ repository. The unique entries of SARS-CoV-2 (142), SARS (221), MERS (123), and overall Coronaviruses (414) were subdivided into the training/testing and independent validation datasets, followed by the extraction of chemical/structural descriptors and fingerprints (17968). The highly relevant features were filtered using the recursive feature selection algorithm. The selected chemical descriptors were used to develop prediction models with Pearson’s correlation coefficients ranging from 0.60 to 0.90 on training/testing. The robustness of the predictive models was further ensured using external independent validation datasets, decoy datasets, applicability domain, and chemical analyses. The developed models were used to predict promising repurposed drug candidates against coronaviruses after scanning the DrugBank. Top predicted molecules for SARS-CoV-2 were further validated by molecular docking against the spike protein complex with ACE receptor. We found potential repurposed drugs namely Verteporfin, Alatrofloxacin, Metergoline, Rescinnamine, Leuprolide, and Telotristat ethyl with high binding affinity. These ‘anticorona' computational models would assist in antiviral drug discovery against SARS-CoV-2 and other Coronaviruses.

## Graphical abstract

**Figure:**
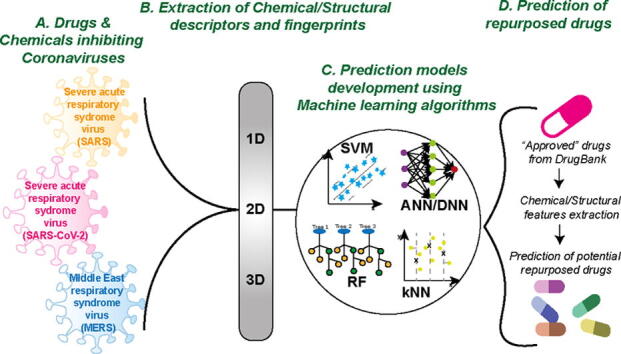

## 1 Introduction

The 21^st^ century has experienced three novel coronavirus (CoV) pandemics caused by the Severe Acute Respiratory Syndrome Virus (SARS), Middle East Respiratory Syndrome Virus (MERS), and Severe Acute Respiratory Syndrome Coronavirus 2 (SARS-CoV-2). The first SARS epidemic from November 2002 till July 2003 led to around 8,000 reported cases, including about 700 deaths worldwide (https://www.who.int/csr/sars/country/table2004_04_21/en/). After about ten years, in June 2012, a second global CoVs outbreak, i.e., MERS, continued until 2016, resulting in around 1,700 confirmed cases, including about 620 deaths globally [1]. The third and ongoing SARS-CoV-2 pandemic, officially declared by WHO in January 2020, has led to around 100 million global cases, including around 3 million deaths as of April 2021.

Coronaviruses are spherically shaped (approx. 125 nm diameter), positive-sense single-stranded RNA viruses [2], [3]. They have been classified into the order *Nidovirales*, family *Coronaviridae*, and subfamily *Orthocoronavirinae*, and have the largest genome ranging from 26 to 32 kb among the RNA viruses. They are further grouped into alpha-coronavirus (α-CoV), beta-coronavirus (β-CoV), gamma-coronavirus (γ-CoV), and delta-coronavirus (d-CoV) based on their genetic as well as antigenic variation [4]. SARS-CoV-2 is an enveloped, positive-sense, unsegmented single-stranded RNA virus that belongs to the genus *Betacoronavirus* [5]. SARS-CoV-2 genome shows 79% and 50% sequence similarity to SARS and MERS genomes, respectively [6]. The CoVs particles majorly consist of four different structural proteins, i.e., surface glycoprotein (S), membrane glycoprotein (M), envelope (E), and nucleocapsid (N) [7], [8] while, some CoVs also encode auxiliary proteins which play remunerate functions. The homotrimeric class I fusion protein, namely S protein, allows the viral membrane to fuse with the host cell surface receptors angiotensin-converting enzyme 2 (ACE2), leading to fusion and viral entry [9], [10] leading to SARS [11]. Additionally, SARS-CoV-2 has been reported to cause systemic infections in the digestive, circulatory, urogenital, and nervous system [12].

Different prophylactic and therapeutic approaches, *viz*., vaccine development, cellular therapies, have been deployed to tackle CoVs diseases. Besides all these strategies, drug repurposing studies, i.e., looking for the efficacy of existing FDA-approved drugs against CoVs, have been very crucial in this regard. Nucleoside analog remdesivir (GS-5734) [13], [14] chloroquine [15], [16], [17] and hydroxychloroquine [18], [19] are effective *in vitro* against SARS, MERS, and SARS-CoV-2. Also, lopinavir/ritonavir (anti-retroviral drugs) against SARS [20], MERS [21], [22], and SARS-CoV-2 [23], [24] are reported to be effective in combination with other drugs such as ribavirin and interferon-β.

Presently, the ongoing SARS-CoV-2 global pandemic requires an urgent need for antiviral therapeutics to control its spread. Lack of effective therapeutics to date necessitates the development of predictive computational tools that can speed up and support the existing/ongoing experimental approaches for drug repurposing. Molecular docking and dynamic simulations based on virtual screening to identify antiviral compounds against SARS-CoV-2 have already been explored in this context [25], [26]. Repurposed drug identification by machine learning techniques (MLTs) based approaches is less explored in CoVs' drug discovery venture to date. The MLTs based predictive algorithms have previously been employed in the development of various antiviral predictors viz., AVPpred [27], AVP-IC_50_ Pred [28], HIVprotl [29], anti-flavi [30], anti-nipah. However, our group recently developed a comprehensive platform for analysis and identification of the epitopes for the CoVs named ‘CoronaVR’ [31]. The input anti-CoVs data in the current study was taken from our recently published comprehensive database of the experimentally validated repurposed drug database named ‘DrugRepV’ [32]. In the current study, we have identified repurposed drug candidates (against SARS-CoV-2, SARS, and MERS) using different MLTs like Support Vector Machine (SVM), Random Forest (RF), k-Nearest Neighbour (KNN), Artificial Neural Network (ANN), and Deep Learning [Deep Neural network (DNN), Artificial Intelligence]. Further, we also predict the effective anti-Corona compounds after scanning the DrugBank repository through the developed predictive models.

## 2 Results

The robust prediction models were developed using various MLTs like SVM, RF, KNN, ANN, and DNN. The efficacies of the training/testing and independent validation dataset were checked using the performance parameters like Mean Absolute Error (MAE), Root Mean Square Error (RMSE), Coefficient of Determination (R^2^), and Pearson’s Correlation Coefficient (PCC or R). The chemical analysis was also performed on the anti-CoVs (SARS, MERS, and SARS-CoV-2) compounds. Further, the drug repurposing was done by scanning the DrugBank through the developed machine learning models.

### 2.1 Feature selection approach

Among the 17,968 descriptors and fingerprints, the 50 best performing features of SARS, MERS, SARS-CoV-2, and overall CoVs were selected, which represented their signatures (Supplementary Table S1). In case of the SARS-CoV-2, the features like ExtFP172 (CDK extended fingerprints), RDF55u (RDF Descriptor, three dimensions (3D)), KRFP504 (Klekota-Roth fingerprint), FP112 (CDK fingerprint), maxdNH (Electrotopological State Atom Type Descriptor, two dimensional (2D)), FP8 (CDK fingerprint), L3i (PaDEL WHIM Descriptor, 3D), E2e (PaDEL WHIM Descriptor, 3D), Km (PaDEL WHIM Descriptor, 3D), ExtFP756 (CDK extended fingerprints), etc. However, for the MERS virus, the descriptors like MOMI-XY, E3p, GraphFP309, P1m, PubchemFP462, TDB10u, minHBint2, KRFPC3596, etc. Likewise, for viruses like SARS and overall CoVs, top-50 features were extracted from the recursive feature selection algorithm (Supplementary Table S1).

### 2.2 Quantitative structure–activity relationship model development

For SARS, various prediction models were developed using the MLTS like SVM, RF, KNN, and ANN. The performance of the training/testing dataset with 198 datasets was calculated using the 10-fold cross-validation (Table 1). The prediction model developed using the training/testing dataset achieved a PCC of 0.92, 0.76, 0.76, and 0.73, from SVM, RF, KNN, ANN, respectively. In contrast, the 23 sequences of the independent validation dataset give an accuracy of 0.90, 0.82, 0.79, and 0.92 correspondingly for the SVM, RF, KNN, and ANN (Table 2). However, the training/testing and independent validation dataset show PCC of 0.59 and 0.23, respectively, for the DNN machine learning (Supplementary Table S2).

**Table 1:** The performance of the Severe Acute Respiratory Syndrome Virus (SARS), Middle East Respiratory Syndrome Virus (MERS), Severe Acute Respiratory Syndrome CoronaVirus 2 (SARS-CoV-2), and Overall Coronaviruses among the training/testing dataset during 10-fold cross validation using Support Vector Machine (SVM), Random Forest (RF), k-Nearest Neighbour (KNN), and Artificial Neural Network (ANN).

Virus	Algorithm	Model Parameters	Dataset	MAE	RMSE	R2	PCC
SARS	SVM	gamma:0.001C:50	T^198^	0.21	0.42	0.82	0.92
	RF	n:100 depth:10 split:5 leaf:1	T^198^	0.49	0.74	0.54	0.76
	KNN	k:9	T^198^	0.50	0.69	0.53	0.76
	ANN	activation:tanh solver:sgd learning:adaptive	T^198^	0.83	0.92	0.14	0.73
SARS- CoV-2	SVM	gamma:0.005C:50	T^127^	0.37	0.58	0.60	0.84
	RF	n:500 depth:12 split:2 leaf:1	T^127^	0.84	0.86	0.15	0.50
	KNN	k:11	T^127^	0.86	1.01	0.04	0.50
	ANN	activation:tanh solver:sgd learning:constant	T^127^	2.46	1.80	0.39	0.62
MERS	SVM	gamma:0.0005C:100	T^110^	0.08	0.30	0.78	0.92
	RF	n:400 depth:8 split:2 leaf:4	T^110^	0.37	0.53	0.16	0.60
	KNN	k:5	T^110^	0.30	0.56	0.29	0.65
	ANN	activation:relu solver:sgd	T^110^	1.04	0.69	0.16	0.49
Overall Coronaviruses	SVM	gamma:0.0005C:500	T^372^	0.81	0.84	0.51	0.73
	RF	n:400 depth:None split:10 leaf:4	T^372^	1.19	1.08	0.31	0.58
	KNN	k:5	T^372^	1.23	1.10	0.28	0.57
	ANN	activation:tanh solver:sgd learning:constant	T^372^	0.95	0.94	0.43	0.68

MAE, Mean absolute Error; RMSE, Root Mean Absolute Error; R2, Coefficient of Determination; PCC, Pearson’s correlation coefficient.

**Table 2:** The performance of the Severe Acute Respiratory Syndrome Virus (SARS), Middle East Respiratory Syndrome Virus (MERS), Severe Acute Respiratory Syndrome CoronaVirus 2 (SARS-CoV-2), and Overall Coronaviruses among the independent validation dataset during 10-fold cross-validation using Support Vector Machine (SVM), Random Forest (RF), k-Nearest Neighbour (KNN), and Artificial Neural Network (ANN).

Virus	Algorithm	Model Parameters	Dataset	MAE	RMSE	R2	PCC
SARS	SVM	gamma:0.001C:50	V^23^	0.20	0.44	0.77	0.90
	RF	n:100 depth:10 split:5 leaf:1	V^23^	0.47	0.69	0.65	0.82
	kNN	k:9	V^23^	0.47	0.69	0.60	0.79
	ANN	activation:tanh solver:sgd learning:adaptive	V^23^	0.26	0.51	0.81	0.92
SARS- CoV-2	SVM	gamma:0.005C:50	V^15^	0.21	0.46	0.81	0.92
	RF	n:500 depth:12 split:2 leaf:1	V^15^	0.90	0.95	0.14	0.50
	kNN	k:11	V^15^	0.52	0.72	0.35	0.67
	ANN	activation:tanh solver:sgd learning:constant	V^15^	2.64	1.62	0.66	0.68
MERS	SVM	gamma:0.0005C:100	V^13^	0.47	0.68	0.69	0.92
	RF	n:400 depth:8 split:2 leaf:4	V^13^	0.74	0.86	0.32	0.74
	kNN	k:5	V^13^	1.16	1.08	0.24	0.69
	ANN	activation:relu solver:sgd	V^13^	0.75	0.87	0.39	0.50
Overall *Coronaviruses*	SVM	gamma:0.0005C:500	V^42^	0.78	0.88	0.53	0.75
	RF	n:400 depth:None split:10 leaf:4	V^42^	1.03	1.02	0.20	0.49
	kNN	k:5	V^42^	1.00	1.00	0.22	0.58
	ANN	activation:tanh solver:sgd learning:constant	V^42^	1.02	1.01	0.39	0.67

MAE, Mean Absolute Error; RMSE, Root Mean Square Error; R2, Coefficient of Determination; PCC, Pearson’s Correlation Coefficient.

The prediction models were also developed for the MERS using 10-fold cross-validation on training/testing and independent validation datasets (Table 1). The training/testing with 110 datasets displayed a PCC of 0.92, 0.60, 0.65, and 0.49, respectively, for the SVM, RF, KNN, and ANN algorithms. While for the 13 independent validation datasets, the MLTs lead to the PCC of 0.92, 0.74, 0.69, and 0.50 correspondingly (Table 2). However, the PCC of the training/testing and independent validation dataset are 0.53 and 0.53, respectively, for the DNN machine learning (Supplementary Table S2).

The SARS-CoV-2 dataset was subdivided into 127 training/testing and 15 independent validation dataset (Table 1). The training/testing dataset shows the PCC of 0.84, 0.50, 0.50, and 0.62, respectively, through the SVM, RF, KNN, and ANN algorithms. However, the independent validation dataset resulted in the PCC of 0.92, 0.50, 0.67, and 0.68 correspondingly on the MLTs (Table 2). The training/testing and independent validation datasets show the PCC of 0.70 and 0.51, respectively, for the DNN machine learning (Supplementary Table S2).

The *Overall* CoVs include unique entries from the SARS, MERS, and SARS-CoV-2 datasets. The overall entries were split into the training/testing and independent validation datasets with 372 and 42 entries *via* the randomization approach available in SciKit library (Table 1). The training/testing dataset provides the PCC of 0.73, 0.58, 0.57, and 0.68, respectively, during 10-fold cross-validation through SVM, RF, KNN, and ANN. In comparison, the independent validation dataset provides the PCC of 0.75, 0.49, 0.58, and 0.67 correspondingly for the MLTs (Table 2). However, the PCC of the training/testing and independent validation dataset are 0.61 and 0.67, respectively, for the DNN machine learning (Supplementary Table S2).

### 2.3 Applicability domain analysis

**Fig. 1:**
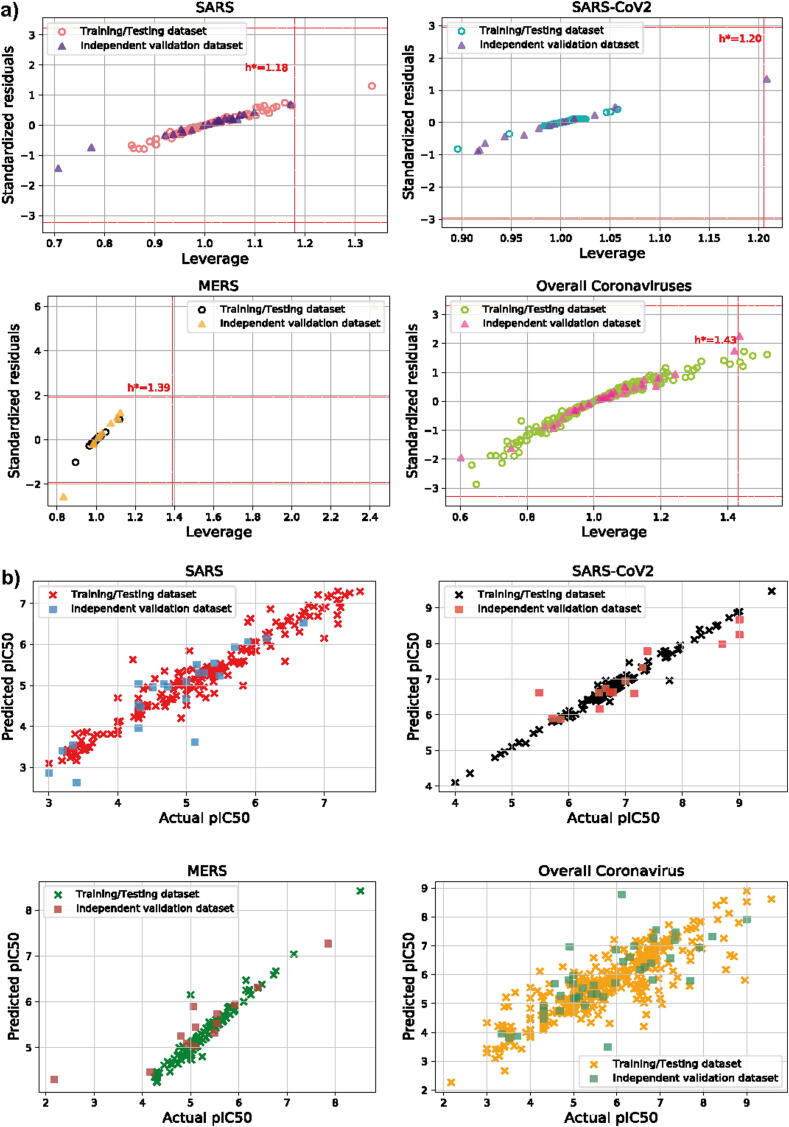
The robustness of the Support Vector Machine models of the Severe Acute Respiratory Syndrome (SARS), Middle East Respiratory Syndrome (MERS), Severe Acute Respiratory Syndrome Coronavirus 2 (SARS-CoV-2), and overall Coronavirus was checked using the a) William’s plot between the leverage and the standardized residuals. b) the plot between the actual and predicted pIC50.

The applicability domain was calculated between the leverage and the standardized residuals among the best performing SVM models. All the models of SVM on the SARS, SARS-CoV-2, MERS, and overall CoVs are highly robust with the leverage (h*) of 1.18, 1.20, 1.39, and 1.43 as shown in Fig. 1**a**. The actual and the predicted pIC50 plots among the SVM models of the SARS, SARS-CoV-2, MERS, and the overall CoVs, also show their robustness, as shown in Fig. 1**b**.

### 2.4 Validation using the decoy set

**Fig. 2:**
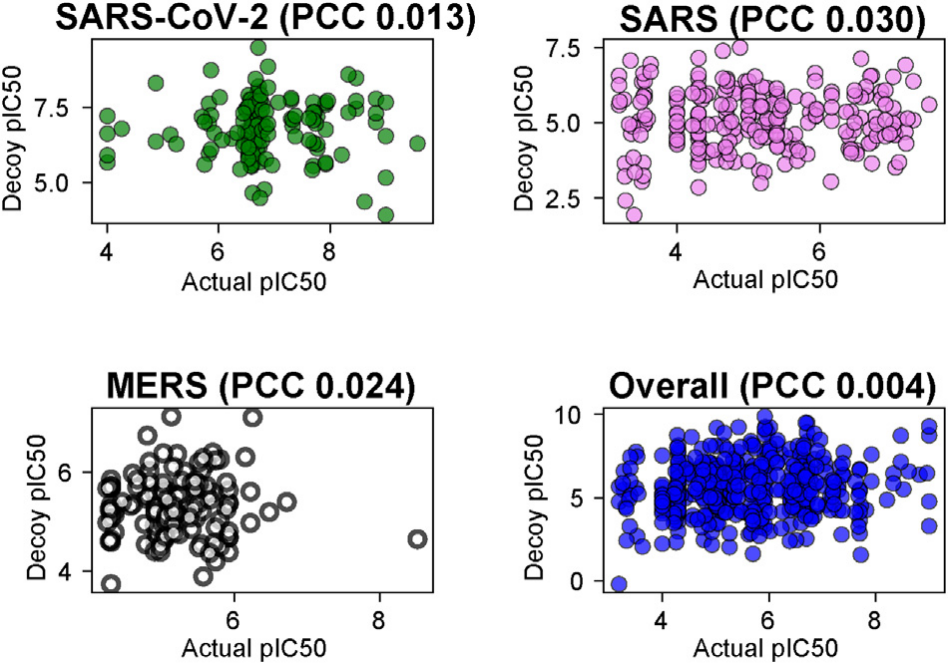
The scatter plot shows the correlation between the actual pIC50 and the predicted pIC50 of the decoy dataset for Severe Acute Respiratory Syndrome Coronavirus 2 (SARS-CoV-2), Severe Acute Respiratory Syndrome (SARS), Middle East Respiratory Syndrome (MERS), and overall coronaviruses.

For all the developed models, the PCC values were calculated for the random decoy sets by comparing the predicted pIC50 of a decoy and its corresponding parent molecule. The SARS decoy dataset shows the PCC of 0.10, 0.08, and 0.03 on sets 1, 2, and 3, respectively. On SARS-CoV-2, we achieved PCC of 0.05, 0.01, and 0.05 on three sets. In the case of MERS, PCC of 0.11, 0.02, and 0.13 was obtained on sets 1, 2, and 3, respectively. The overall CoVs show the PCC of 0.06, 0.01, and 0.004 on set 1, 2, and 3, respectively (Fig. 2 and Supplementary Table S9).

### 2.5 Chemical diversity of anti-Coronaviruses molecules

**Fig. 3:**
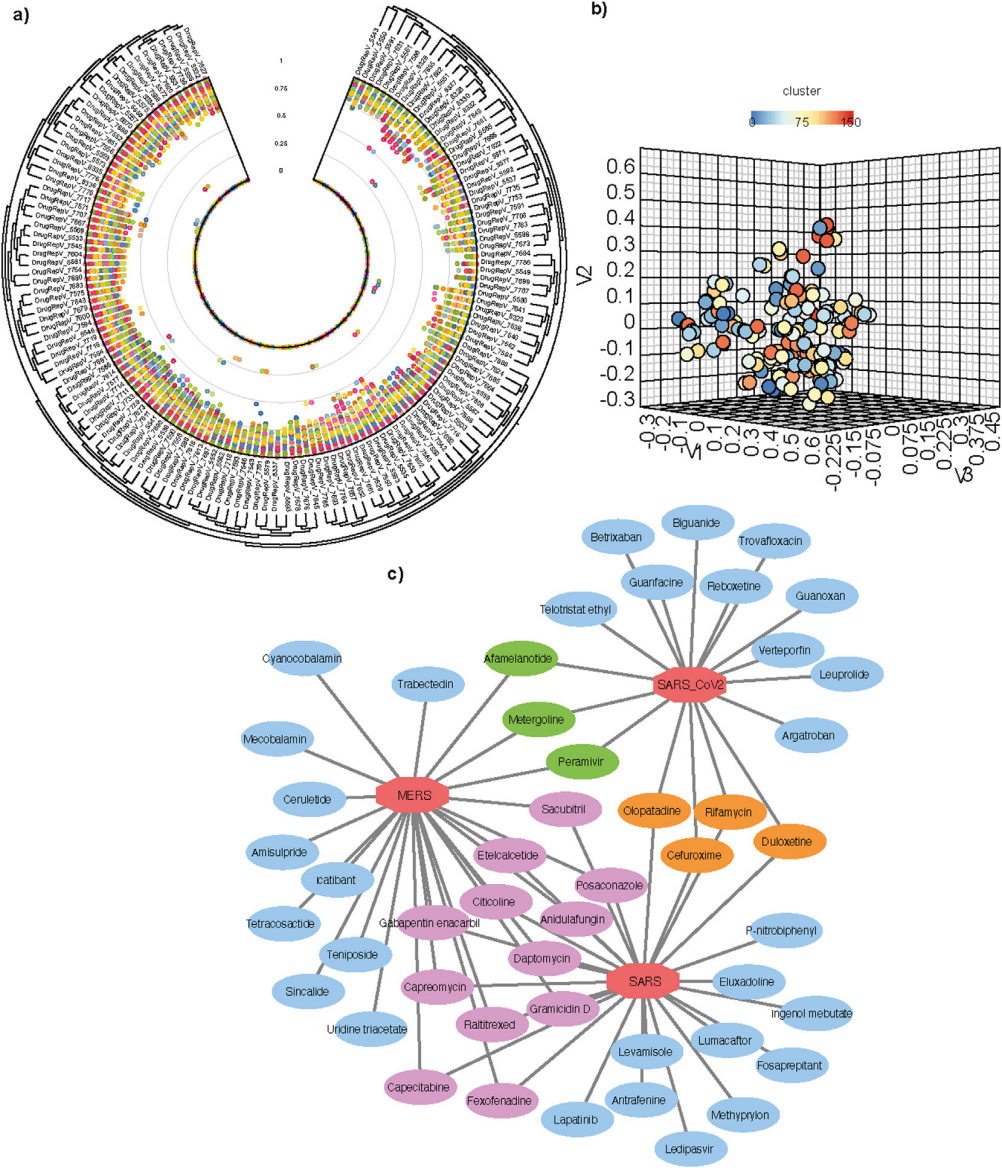
The chemical analysis of the Severe Acute Respiratory Syndrome CoronaVirus 2 (SARS-CoV-2) inhibitors a) The hierarchical clustering of the SARS-CoV-2 is depicted using the circular plots, b) The 3-dimensional multiscaling plot among the SARS-CoV-2 inhibitors. c) Chemical network showing the status of top-10 predicted repurposed drugs against Coronaviruses (SARS, SARS-CoV-2, and MERS). Blue color of the drug shows the predicted repurposed drugs unique to single virus, green color depicts the common repurposed drugs between SARS-CoV-2 and MERS, orange color shows the common repurposed rugs between SARS and SARS-CoV-2, while the pink color shows the common drug between the SARS and MERS. (For interpretation of the references to color in this figure legend, the reader is referred to the web version of this article.)

Binning clustering of 221 anti-SARS compounds with a similarity cut-off of 0.60 produced 101 bins. Similarly, binning clustering of 123 anti-MERS compounds with a similarity cut-off of 0.60 produced 53 bins. Futhermore, binning clustering of 142 anti-SARS-CoV-2 compounds with a similarity cut-off of 0.60 produced 131 bins. Multidimensional scaling at 3D showed the diversity of the anti-SARS-CoV-2 compounds in the chemical space Fig. 3**a**. Hierarchical clustering of the anti-SARS-CoV-2 compounds using the single linkage method provided the hierarchy of compound clusters provided in the form of circular plots, which shows high chemical diversity in among them Fig. 3**b**. However, the 3D multidimensional scaling and the hierarchical clustering of SARS and MERS are shown in Supplementary Fig. S1. The 3D multidimensional scaling shows that all the anti-corona compounds are highly dissimilar in chemical structures. The anti-SARS-CoV-2 compounds are in more chemical diversity, followed by the anti-MERS and the anti-SARS.

### 2.6 Prediction of promising repurposed anti-Coronavirus drug candidates

The best performing SVM predictors were used to identify the repurposed drug candidates against SARS, MERS, and SARS-CoV-2 (Fig. 3**c,** Supplementary Figs. S2-S4). For the SARS virus, the drugs with high efficacies are Antrafenine, Methyprylon, Fosaprepitant, Ledipasvir, Raltitrexed, Lumacaftor, Eluxadoline, Ingenol mebutate, Lapatinib, Sacubitril, and Capreomycin with IC_50_ of 0.01, 0.02, 0.02, 0.03, 0.05, 0.05, 0.05, 0.05, 0.05, 0.07, and 0.07 μM respectively (Supplementary Table S3). However, for MERS, the best-performing drugs Cyanocobalamin, Ceruletide, Teniposide, Trabectedin, Sincalide, Tetracosactide, Icatibant, Amisulpride, Tipranavir, Gabapentin enacarbil, and Peramivir has IC_50_ of 0.02, 0.03, 0.04, 0.04, 0.04, 0.06, 0.06, 0.06, 0.07, and 0.08 μM respectively (Supplementary Table S4). In case of the SARS-CoV-2, the drugs Verteporfin, Argatroban, Reboxetine, Guanfacine, Telotristat ethyl, Betrixaban, Leuprolide, Trovafloxacin, Peramivir, Salmeterol, Oxybuprocaine, and Warfarin are predicted drugs with high performance of IC_50_ of 0.0003, 0.0004, 0.0005, 0.0007, 0.0007, 0.0008, 0.0008, 0.0009, 0.0009, 0.0010, 0.0011, 0.0011, and 0.0012 μM respectively (Table 3).

**Table 3:** Table showing the top hits of the predicted repurposed drug candidates against Severe Acute Respiratory Syndrome Coronavirus 2 (SARS-CoV-2) with the information like DrugBank ID, Drug Name, Primary indication, Predicted pIC50, and testing status.

DrugBank ID	Drug Name	Primary indication	Predicted pIC50	Status
DB00007	Leuprolide	Prostate cancer; Central precocious puberty (CPP)	9.093	Not Yet tested
DB00014	Goserelin	Prostate cancer	8.641	Not Yet tested
DB00050	Cetrorelix	Premature LH surge	8.342	Not Yet tested
DB00148	Creatine	Dietary shortage or imbalance	8.594	Not Relevant
DB00206	Reserpine	Hypertension; Psychotic disorder	8.728	Clinical trial - Observational
DB00234	Reboxetine	Clinical depression	9.308	Not Yet tested
DB00248	Cabergoline	Hyperprolactinemic disorders and Parkinsonian Syndrome	8.370	Not Yet tested
DB00266	Dicoumarol	Coagulation disorders	8.357	Not Yet tested
DB00278	Argatroban	Coagulation disorders	9.357	Clinical trial - Interventional
DB00289	Atomoxetine	Attention deficit hyperactivity disorder (ADHD)	8.563	Not Yet tested
DB00331	Metformin	Diabetes	8.498	Clinical trial - Interventional
DB00381	Amlodipine	Hypertension	8.363	Clinical trial - interventional
DB00460	Verteporfin	Subfoveal choroidal neovascularization	9.556	Not Yet tested
DB00470	Dronabinol	Anorexia	8.604	Computational
DB00476	Duloxetine	Depressive Disorder	8.736	Not Yet tested
DB00486	Nabilone	Nausea and vomiting	8.535	Not Yet tested
DB00536	Guanidine	Muscle weakness; Myasthenic syndrome of Eaton-Lambert	8.640	Not Yet tested
DB00579	Mazindol	Obesity	8.705	Not Yet tested
DB00589	Lisuride	Parkinson's Disease	8.422	Computational
DB00590	Doxazosin	Benign prostatic hypertrophy	8.668	Clinical trial - Observational
DB00641	Simvastatin	Cardiovascular agents	8.404	Clinical trial - Interventional
DB00644	Gonadorelin	Gonadotropes of the anterior pituitary	8.581	Not Yet tested
DB00666	Nafarelin	Central precocious puberty	8.621	Computational
DB00682	Warfarin	Coagulation disorders	8.924	Clinical trial - Observational
DB00685	Trovafloxacin	For treatment of infections caused by microorganisms	9.041	Computational
DB00706	Tamsulosin	Benign prostatic hyperplasia	8.890	Not Yet tested
DB00738	Pentamidine	Pneumonia	8.592	Not Yet tested
DB00768	Olopatadine	Allergic conjunctivitis	8.341	Not Yet tested
DB00776	Oxcarbazepine	Partial seizures	8.402	Not Yet tested
DB00778	Roxithromycin	Respiratory tract; Urinary and soft tissue infections	8.459	Not Relevant
DB00807	Proparacaine	Ophthalmic anesthetic	8.810	Clinical trial - Observational
DB00887	Bumetanide	Edema associated with congestive heart failure, hepatic and renal disease	8.548	Clinical trial - Observational
DB00892	Oxybuprocaine	Used to temporarily numb the front surface of the eye	8.945	Not Yet tested
DB00914	Phenformin	Type 2 diabetes mellitus	8.443	Computational
DB00938	Salmeterol	Asthma; Chronic obstructive pulmonary disease	8.976	Not Yet tested
DB00955	Netilmicin	Bacteremia; Septicaemia; Respiratory tract infections	8.314	Not Yet tested
DB01018	Guanfacine	Attention deficit hyperactivity disorder (ADHD)	9.152	Clinical trial - Observational
DB01079	Tegaserod	Irritable bowel syndrome	8.521	Not Yet tested
DB01082	Streptomycin	Tuberculosis	8.887	Computational
DB01089	Deserpidine	Hypertension	8.555	Not Yet tested
DB01110	Miconazole	Fungal infections	8.626	Not Relevant
DB01131	Proguanil	Malaria	8.600	Computational
DB01180	Rescinnamine	Hypertension	8.921	Not Yet tested
DB01283	Lumiracoxib	Osteoarthritis	8.464	Not Yet tested
DB01418	Acenocoumarol	Thromboembolic disease	8.800	Clinical trial - Observational
DB01764	Dalfopristin	Bacterial infections	8.595	Not Yet tested
DB03615	Ribostamycin	NA	8.395	Not Yet tested
DB04840	Debrisoquine	Hypertension	8.713	Not Yet tested
DB04864	Huperzine A	Alzheimer's disease	8.852	Not Yet tested
DB04868	Nilotinib	Leukemia	8.442	Experimental
DB04931	Afamelanotide	Phototoxicity	8.492	Not Yet tested
DB06145	Spiramycin	Bacterial infections	8.634	Computational
DB06614	Peramivir	Influenza A/B virus	9.018	Computational
DB06616	Bosutinib	Chronic myelogenous leukemia (CML)	8.489	Experimental
DB06636	Isavuconazonium	Aspergillosis; Mucormycosis	8.313	Clinical trial - Interventional
DB06663	Pasireotide	Cushing’s disease	8.480	Not Yet tested
DB06784	Gallium citrate Ga-67	Hodgkin's disease, lymphoma, and bronchogenic carcinoma	8.419	Not Yet tested
DB08912	Dabrafenib	Melanoma	8.788	Computational
DB08916	Afatinib	Metastatic non-small cell lung cancer	8.391	Not Yet tested
DB08943	Isoconazole	NA	8.577	Not Yet tested
DB08995	Diosmin	NA	8.394	Clinical trial - Interventional
DB09084	Benzydamine	Analgesic and anti-inflammatory treatment	8.720	Not Yet tested
DB09125	Potassium citrate	Renal tubular acidosis	8.394	Not Yet tested
DB09157	Carbon dioxide	Insufflation gas for minimal invasive surgery	8.619	Not Relevant
DB09335	Alatrofloxacin	NA	8.862	Not Yet tested
DB11512	Dihydrostreptomycin	NA	8.830	Not Yet tested
DB11574	Elbasvir	HCV genotypes 1 or 4	8.724	Computational
DB11753	Rifamycin	Traveller's Diarrhea	8.359	Computational
DB11827	Ertugliflozin	Type 2 diabetes	8.522	Not Yet tested
DB11828	Neratinib	Breast cancer	8.401	Not Yet tested
DB12095	Telotristat ethyl	To reduce serotonin levels	9.135	Not Yet tested
DB12364	Betrixaban	Venous thromboembolism (VTE)	9.116	Computational
DB12500	Fedratinib	Myelofibrosis	8.438	Not Yet tested
DB12615	Plazomicin	Complicated Urinary Tract Infections (cUTI)	8.348	Not Yet tested
DB13100	Biguanide	NA	9.221	Not Yet tested
DB13211	Guanoxan	NA	9.694	Not Yet tested
DB13520	Metergoline	NA	8.704	Not Yet tested
DB13680	Naftazone	NA	8.342	Not Yet tested
DB14575	Eslicarbazepine	NA	8.318	Not Yet tested
DB14753	Hydroxystilbamidine	Nonprogressive blastomycosis of the skin and other mycoses	8.314	Not Yet tested

### 2.7 Molecular docking

**Fig. 4:**
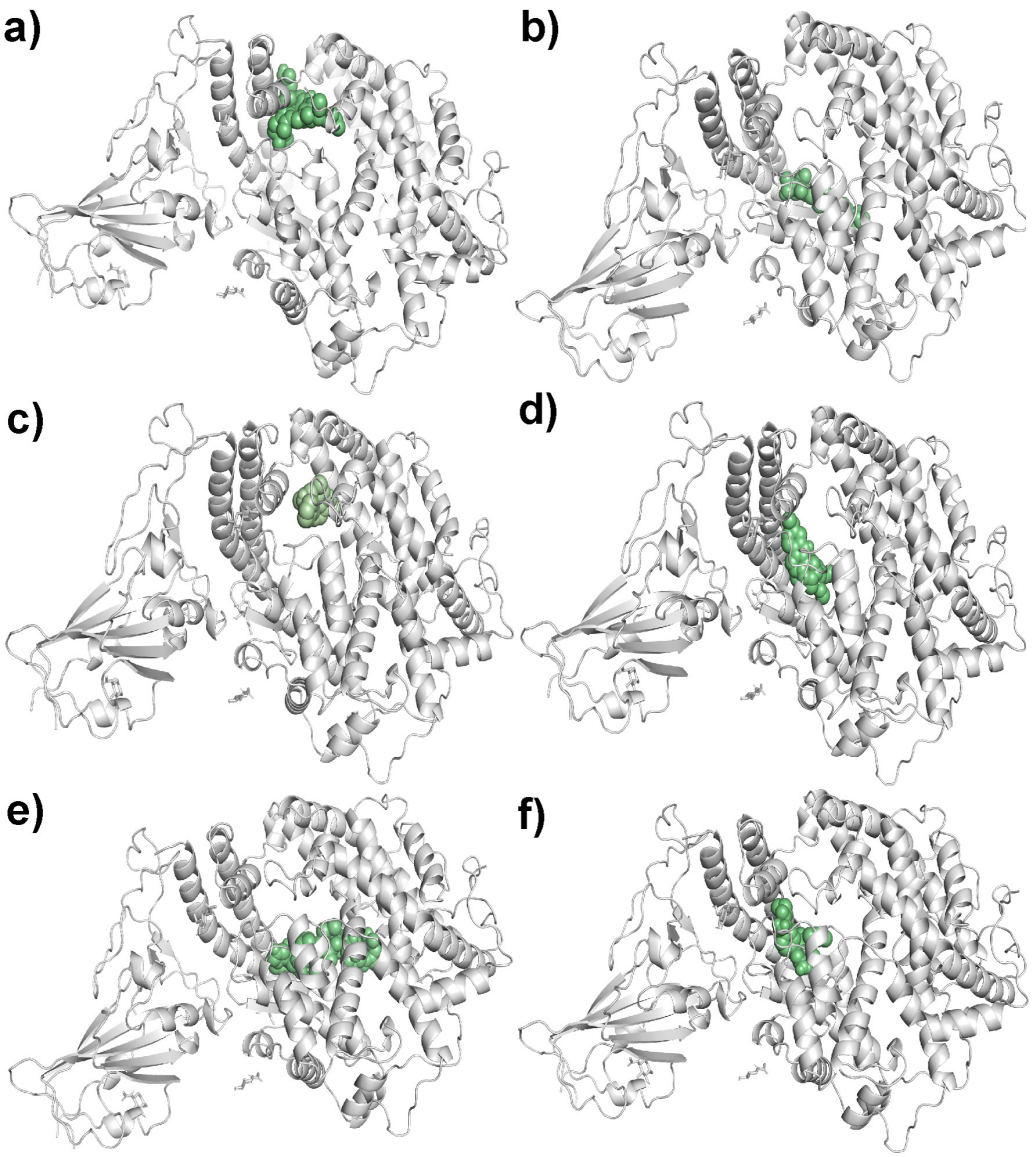
The ligands a) Verteporfin, b) Alatrofloxacin, c) Metergoline, d) Rescinnamine, e) Leuprolide, and f) Telotristat ethyl binding the SARS-CoV-2 S-protein. (SARS-CoV-2 S-protein in ribbon diagram with grey color and ligand molecule in green color sphere). (For interpretation of the references to color in this figure legend, the reader is referred to the web version of this article.)

**Fig. 5:**
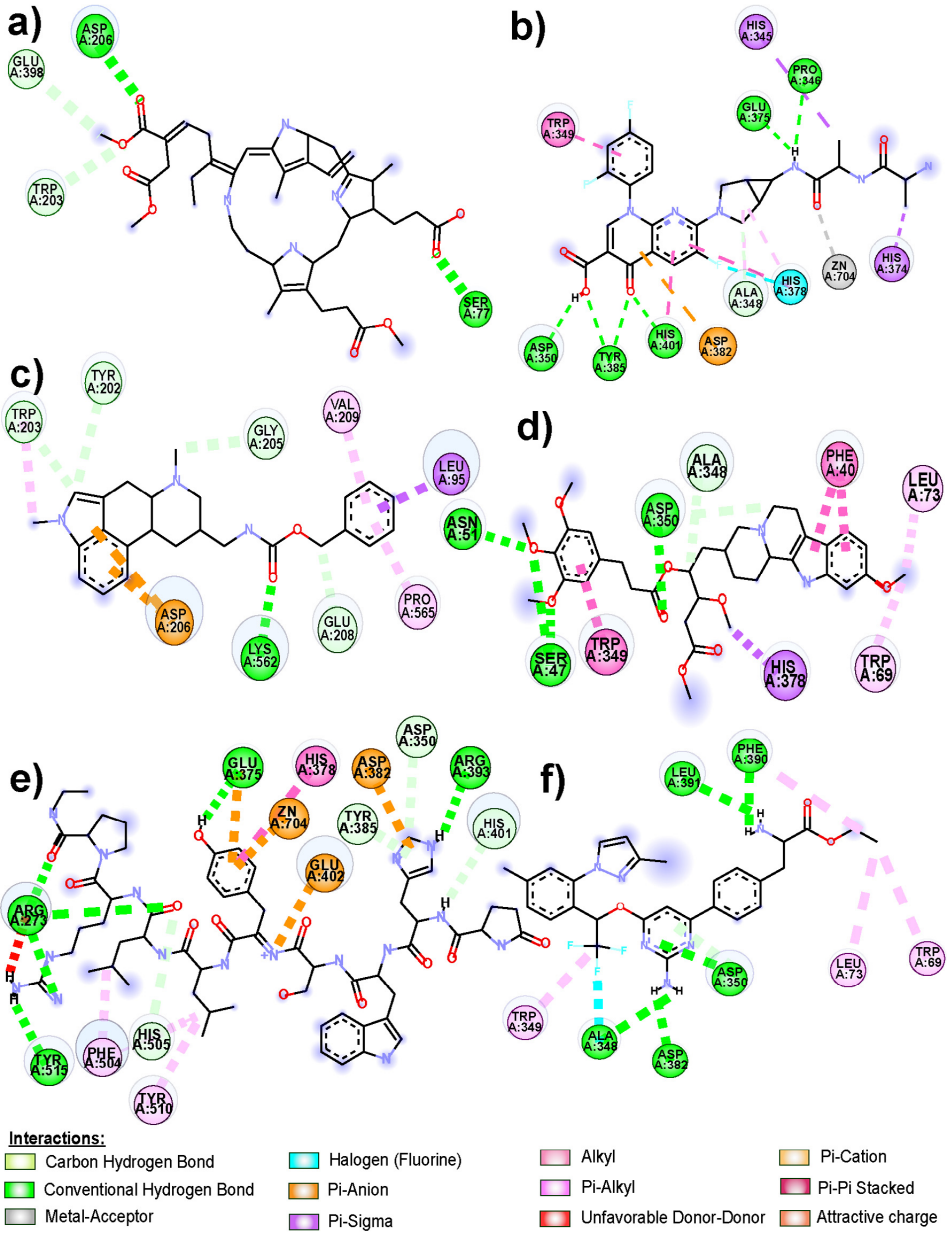
Two-dimensional representation of molecular interactions of a) Verteporfin, b) Alatrofloxacin, c) Metergoline, d) Rescinnamine, e) Leuprolide, and f) Telotristat ethyl with the S-protein of SARS-CoV-2.

The molecular docking technique is highly beneficial for understanding the protein-ligand interactions and bond lengths among them. We have selected the top 20 compounds out of 80 predicted molecules for SARS-CoV-2 based on their predicted high pIC50 value. These compounds were docked sequentially on SARS-CoV-2 S protein (PDB: 6lzg) to calculate their best binding affinity in Kcal/mol. The detailed result of their binding affinities are shown in Supplementary Table S10. Analysis of binding affinity showed that 15 out of 20 compounds have binding energies ranging from −6.8 Kcal/mol to −9.5 Kcal/mol. These 15 compounds were selected for the interaction with SARS-CoV-2 S-protein (PDB: 6LZG), and their comprehensive list is represented in Table 4. Additionally, 06 molecules Verteporfin, Alatrofloxacin, Metergoline, Rescinnamine, Leuprolide, and Telotristat ethyl with binding energy ranging from −8.0 Kcal/mol to −9.5 Kcal/mol and their interacting residues are displayed in Fig. 4, Fig. 5.

**Table 4:** Table represents the ligand, binding affinity, Root Mean Square Deviation (RMSD) value (Å), interacting residues, bond length (Å), type of interactions, as well as interacting domain of Spike protein. N-Terminal Domain (NTD), C-Terminal Domain (CTD), Receptor Binding Domain (RBD)

DrugBank ID	Ligand	Affinity (kcal/mol)	RMSD (Å)	Interacting residues	Bond length(Å)	Interactions	Interacting domain
DB00460	Verteporfin	−9.5	0	SER-77 TRP-203 ASP-206 GLU-398	2.50 3.44, 3.49 2.57 3.67	Hydrogen Bond Carbon-Hydrogen Bond	NTD / CTD (RBD)
DB09335	Alatrofloxacin	−9.1	0	HIS-345 PRO-346 ALA-348 TRP-349 ASP-350 HIS-374 GLU-375 HIS-378 ASP-382 HIS-401 ZN-704	3.98 2.43 3.74, 5.44 5.08, 5.10 2.15 2.34, 3.75 2.80 2.43, 3.43, 4.93, 5.39 4.85 2.82, 4.84	Hydrogen Bond Carbon-Hydrogen Bond Alkyl Bond Pi-Alkyl Metal-Acceptor Pi-Anion	CTD (RBD)
DB13520	Metergoline	−8.8	0	LEU-95 TYR-202 TRP-203 GLY-205 ASP-206 GLU-208 VAL-209 LYS-562 PRO-565	3.43 3.32 3.56, 4.55 3.23 3.31, 3.93 3.36 4.13 2.90, 2.94 4.53	Hydrogen Bond Carbon-Hydrogen Bond Pi-Alkyl Pi-Anion	NTD / CTD (RBD)
DB01180	Rescinnamine	−8.5	0	PHE-40 SER-47 ASN-51 TRP-69 LEU-73 ALA-348 TRP-349 ASP-350 HIS-378	4.98, 5.52 1.94, 2.67 2.52 5.08 4.53 3.09 3.80, 4.61 1.90, 3.77	Hydrogen Bond Carbon-Hydrogen Bond Alkyl Bond	NTD / CTD (RBD)
DB00014	Goserelin	−8.5	0	ASP-350 ASP-382 ARG-393 ASN-394 HIS-401 GLU-402 ARG-514	2.10 2.40 2.10 2.70 2.80 2.80 2.80	NA	CTD (RBD)
DB00007	Leuprolide	−8.2	0	ARG-273 ASP-350 GLU-375 HIS-378 ASP-382 TYR-385 ARG-393 HIS-401 GLU-402 PHE-504 HIS-505 TYR-510 TYR-515 ZN-704	1.40, 2.00, 2.50, 2.88, 3.37 3.44 2.20, 4.41 3.50, 5.20 4.26 3.00 2.29 2.73 2.05, 2.32, 2.43, 4.00, 4.74 5.03 4.42 2.44	Hydrogen Bond Carbon-Hydrogen Bond	NTD / CTD (RBD)
DB12095	Telotristat ethyl	−8	0	TRP-69 LEU-73 ALA-348 TRP-349 ASP-350 ASP-382 PHE-390 LEU-391	5.06 4.95 2.93, 3.39 3.97, 4.12 2.17, 2.72 2.14 2.23, 4.91 2.82	Hydrogen Bond Alkyl Pi-Alkyl Pi-Donor Hydrogen Bond	NTD / CTD (RBD)
DB11512	Dihydrostreptomycin	−7.6	0	GLN-102 TRY-202 TRP-203 GLY-205 ASP-206 GLU208 ARG-514	2.81 2.74, 2.91 2.82 2.49, 3.33 2.49 2.17, 2.60 1.53	Hydrogen Bond Carbon-Hydrogen Bond Alkyl Pi-Alkyl Metal-Acceptor Pi-Anion	NTD / CTD (RBD)
DB00706	Tamsulosin	−7.3	0	SER-43 TRP-349 ASP-350 ARG-393	2.23, 2.29 4.56 3.71 4.68	Hydrogen Bond Carbon-Hydrogen Bond Pi-Alkyl Pi-Pi Stacked	NTD / CTD (RBD)
DB04840	Debrisoquine	−7.3	0	LEU-95 ASP-206 GLU-208 VAL-209 LYS-562 PRO-565	3.43 2.32, 2.42, 4.93 4.11 5.05 4.65	Hydrogen Bond Carbon-Hydrogen Bond Pi-Alkyl Attractive Charge	NTD / CTD (RBD)
DB00579	Mazindol	−7.2	0	LEU-95 ALA-99 ASP-206 LYS-562	5.12 3.88, 5.03 2.52 3.72, 4.76, 5.12	Hydrogen Bond Alkyl Pi-Alkyl	NTD / CTD (RBD)
DB04864	Huperzine A	−7.1	0	PHE-40 TRP-69 LEU-73 PHE-390 LEU-391 ARG-393	4.95 4.86 4.37 2.11, 4.14, 4.72 5.06 3.08, 5.35, 8.40	Hydrogen Bond Carbon-Hydrogen Bond Alkyl Bond	NTD / CTD (RBD)
DB09084	Benzydamine	−7.1	0	ASP-382 PHE-390 ARG-393 ASN-394	8.30 3.79 4.78 2.69	Hydrogen Bond Carbon-Hydrogen Bond Pi-Alkyl	CTD (RBD)
DB13211	Guanoxan	−7	0	LEU-95 ASP-206 VAL-209 ALA-396 PRO-565	3.70 2.17, 2.98 2.44 4.46	Hydrogen Bond Pi-Alkyl	NTD / CTD (RBD)
DB00476	Duloxetine	−6.8	0	LEU-95 GLN-98 GLU-208 VAL-209 LYS-562 PRO-565	3.44, 4.66 2.79 2.38, 3.18 4.18, 4.81	Hydrogen Bond Carbon-Hydrogen Bond	NTD / CTD (RBD)

Interaction analysis of Verteporfin revealed 03 interactions with the N-terminal domain (NTD) and 01 interaction with the C-terminal domain (CTD) of the SARS-CoV-2 S-protein complex with ACE2 receptor. These interactive residues were SER-77, TRP-203, ASP-206, and GLU-398, which showed the conventional hydrogen bond and carbon-hydrogen bond as shown in Fig. 5, along with their bond lengths of 2.50(Å), 3.44(Å), 2.57(Å), and 3.60(Å), respectively. The receptor-ligand complex formed between Alatrofloxacin and SARS-CoV-2 S-protein showed 12 interactions with the CTD/Receptor binding domain (RBD) of SARS-CoV-2 S-protein complexed with the ACE2 receptor Fig. 5**.** Apart from this, Metergoline shows 09 interactions, out of which 07 interactions belong to the NTD of SARS-CoV-2 S-protein complexed with ACE2 receptor. Further, Rescinnamine shows 09 interactions, out of which 06 interactions occur in the NTD, and the remaining 03 belong to the CTD of SARS-CoV-2 S-protein complexed with ACE2 receptor. Additionally, Leuprolide and Telotristat ethyl showed 14 and 08 interactions, respectively. In leuprolide, 13 out of 14 interactions and in Telotristat ethyl, 06 among the 08 interactions occurred in the CTD/RBD of SARS-CoV-2 S-protein complexed with ACE2 receptor. Table 4 represents the interacting residues, interacting domain of the protein, type of interactions, as well as bond length of the 06 ligands mentioned above.

### 2.8 Status of the predicted repurposed drugs in literature

Apart from performing the cross-validation, internal validation, and applicability domains, we also checked the literature to find support for the experimental validation of our predicted repurposed drugs. For the same, we searched the predicted drugs from our pipelines with the (if provided) inhibition efficiencies reported in the literature through *in vivo*, *in vitro*, and computational approaches (Supplementary Fig. S5). The detail of the top hits predicted from our pipeline, DrugBank ids, drug name, primary indication, and testing status are provided in Table 3.

From predicted drugs for the SARS-CoV-2, 17 drugs are already found in clinical trials like Argatroban, Metformin, Amlodipine, etc. (Table 3). Out of 17 drugs in clinical trials, 06 are reported as interventional studies (Argatroban, Metmorfin, Amlodipine, Simvastatin, Isavuconazium, and Diosmin), 07 are in observational studies (Reserpine, Doxazosin, Warfarin, Proparacaine, Bumetanide, Guanfacine, and Acenocoumarol), and 04 are in clinical studies (but that are not relevant for SARS-CoV-2 treatments). Further, some drugs are also predicted through computational approaches (docking, simulations, etc.) like Lovastatin, Dronabinol, Lisuride, etc. (Table 3). However, some drugs also validated through *in vivo* studies, e.g., Nilotinib showing inhibition win Vero‐E6 cells and Calu‐3 cells with EC_50_ of 1.44 μM and 3.06 μM, respectively, while Bosutinib shows EC_50_ of 2.45 ± 0.12 μM for SARS-CoV-2. Thus, this analysis demonstrates the robustness of our prediction algorithm, which further suggests that the predicted drugs will show promising results against the SARS-CoV-2.

## 3 Discussion

Currently, the world is facing the crisis of SARS-CoV-2 infection, which has led to millions of deaths. Apart from present pandemics of the SARS-CoV-2, other CoVs like SARS and MERS also caused various epidemics/pandemics in past years [33]. Numerous researchers around the world are focusing on developing drugs against the SARS-CoV-2. Drug development is a very complex and time-consuming process. However, in the current scenario of the SARS-CoV-2 pandemic, the need for effective antiviral drugs is critical. In this regard, computational interventions would be an essential step to speed up the research. Researchers have already used different computational approaches to find potential drugs against SARS-CoV-2 infection. To mention a few, Chen TF *et al.*, have developed a drug database, DockCoV2, for SARS-CoV-2 which focuses on predicting the binding affinity of FDA-approved and Taiwan National Health Insurance drugs [46]. Another web server, DockThor-VS, developed by Guedes IA *et al.*, provides a virtual screening (VS) platform with curated structures of potential therapeutic targets from SARS-CoV-2 incorporating genetic information relevant to non-synonymous variations [47]. In another study, Li R *et al.,* used network pharmacology-based computational analyses to understand and characterize the binding capacity, biological functions, pharmacological targets, and therapeutic mechanisms of niacin in colorectal cancer (CRC)/COVID-19 [48]. Again, Kumar A *et al.,* have used a cheminformatics approach to create different datasets and analyzed scaffold diversity to predict the SARS-CoV-2 inhibitors [49]. Recently, Beck B *et al*., used a pre-trained deep learning-based drug-target interaction model called molecule transformer-drug target interaction (MT-DTI) to identify commercially available drugs that could act on SARS-CoV-2 proteins [50]. Further, Zhou Y *et al*. group published their work of integrative network-based systems pharmacology methodology for rapid identification of repurposable drugs and drug combinations for the potential treatment of 2019-nCoV/SARS-CoV-2 [51]. Mainly the inhibitors were designed against the main protease (M^pro^) of SARS-CoV-2 using *in-silico* molecular docking approach. However, the machine learning based approaches are less explored to predict the drugs against SARS-CoV-2 infection.

MLTs based methods using the experimentally validated chemicals/drugs for anti-CoVs activity are lacking. The current study is focused on predicting the efficient and novel drug repurposed candidates for the CoVs, SARS-CoV-2, MERS, and SARS. We extracted the experimentally validated drugs/compounds tested for antiviral activities for CoVs from the ‘DrugRepV’ database. To develop the prediction algorithm, we explored 17,968 chemical and structural descriptors (one dimensional 1D, 2D, and 3D) as well as fingerprints. For the prediction algorithm, we used highly robust methods like feature selection, internal and external validation, MLTs, and applicability domains. Among all MLTs used in developing the predictive models, the SVM outperformed the RF, KNN, ANN, and DNN. The PCC of the SVM model of the CoVs, *i.e.*, SARS, SARS-CoV-2, MERS, and overall ranges from 0.73 to 0.92 on the training/testing datasets. However, the independent validation datasets performed equally well.

Further, the robustness of the model was cross-checked by plotting the applicability domain, and actual vs. predicted pIC50 values. William's plots are used to calculate the applicability of the predictive models and confer the robustness of all the models. Likewise, the analysis of the actual vs. predicted plots also validated the robustness of our models. We have also checked the robustness of the model by using external validation datasets and decoy sets. Using the external validation datasets, we achieved PCCs ranging from 0.60 to 0.90. In comparison, the decoy datasets have PCCs from 0.004 to 0.09. In earlier studies also, the decoy sets had low efficiency compared to corresponding developed models demonstrating the robustness of our computational models for each [30], [41].

Chemical clustering is often used to understand the distribution of compounds in the chemical space. Binning clustering method aggregates chemical compounds to a user-defined similarity cutoff. Here a Tanimoto coefficient (Tc) (proportion of the features shared between two compounds divided by their union) of 0.60 was used. The Tc ranges from 0 to 1, where a higher value indicates the greater similarity of the compounds under investigation. So, using a Tc of 0.60 joined the compounds with 0.60 or higher similarity values together into multiple clusters. As there are many clusters present per ‘anti-corona’ compound groups, the compounds are well dispersed in the chemical space. The multidimensional scaling (MDS) uses the classical multidimensional scaling ‘cmdscale’ function implemented in R and takes a matrix of ‘item to item’ distances as input. Each item is assigned with a coordinate, and the ‘item to item’ distances are then displayed in 2D and 3D scatter plots. The MDS plots generated in the analysis showed that each group of ‘anti-SARS’, ‘anti-MERS’, ‘anti-SARS-CoV-2′ as well as the overall ‘anti-corona’ compounds are well dispersed in the 2D and 3D chemical space. On the other hand, the hierarchical clustering uses the ‘hclust’ function of R and requires a distance matrix input of ‘*all-against-all*’ compound distances. The ‘*all-against-all*’ distance matrix is generated by subtracting the Tc similarity measure from one (1-Tc). Both the hierarchical clustering circular plots generated in the analysis show that the anti-corona compounds are highly dissimilar in their structural features.

Since, drug development is a very complex and time-consuming process, from the start of the SARS-CoV-2 pandemic, several research groups have been trying to identify efficient repurposed drug candidates *via* computational, *in vitro*, and *in vivo* studies. So our developed computational predictive models were used to identify the repurposed drug candidates from the “approved” drug category of the DrugBank database. Further, we checked the predicted repurposed drug candidates using our pipeline, which have been already validated in the literature. Interestingly, we found that a few top hits from our study have been efficiently validated. Thus, it further confirms the robustness of our predictive pipeline. Among the top 10 drug candidates for the SARS-CoV-2 virus with the lowest IC_50_ *i.e.*, Verteporfin has been already validated as the potential ACE2 inhibitor in the *in vitro* and mouse model [34], which has primarily been used to treat age-related degeneration [35], and various types of cancers like prostatic cancer, breast cancer, etc [36]. The Guanfacine drug, which is primarily used to treat Attention Deficit Hyperactivity Disorder (ADHD), is already in use to treat Delirium condition in COVID-19 patients [37]. Likewise, the Trovafloxacin drug, which is a broad-spectrum antibiotic, has been predicted to be an efficient Main protease (M^pro^) inhibitor in a docking study done by Gimeno A, *et al.* [38]. The Argatroban drug, which was earlier used as a thrombin inhibitor also shows promising inhibition against SARS-CoV-2 [39]. The Reboxetine drug, which was initially used to treat clinical depression, shows promising results in the *in vitro* study with ΔG_binding_(kcal/mol) of −8.86 and inhibiting M^Pro^ [40]. Therefore, the repurposed drug candidates predicted by our pipeline could be beneficial to speed up the research in the field of CoVs inhibitors.

Molecular docking and molecular dynamics methods are used as a well-reasoned strategy that provides valuable insights regarding the physicochemical properties of molecules of interest. It also provides the information about the interaction and reactivity of the molecules as potential drug candidates [42]. Few literature reports have identified the repurposed drugs that targets SARS-CoV-2 Spike protein [43], [44], [45]. Current study identifies 06 ligands molecules with high binding affinity, *i.e.* Verteporfin, Alatrofloxacin, Metergoline, Rescinnamine, Leuprolide, Telotristat ethyl against the SARS-CoV-2 S-protein complex with ACE receptor. We found the binding affinity of Metergoline and Rescinnamine, *i.e.*, −8.8 Kcal/mol and −8.5 Kcal/mol, respectively in this study. These findings correspond with the previous study of Chen T-F. *et al.*, which showed the docking scores of −8.4 and −7.5, for Metergoline and Rescinnamine respectively, against SARS-CoV-2 Spike-RBD [46]. Therefore, the present work can contribute to identify the efficacious repurposed drugs against SARS-CoV-2 through computational approaches.

Leveraging this we have developed an AI and MLT based predictor named ‘anticorona’ which includes modules of predictive models for CoVs including SARS-CoV-2, SARS, and MERS, with high performance. We have also ensured the robustness of the predictive models using i) external independent validation datasets, ii) decoy datasets, iii) applicability domain, and iv) chemical analyses. The developed models were used to predict promising repurposed drug candidates against CoVs after scanning the DrugBank. Top predicted molecules for SARS-CoV-2 were further validated by molecular docking against the spike protein complex with ACE receptor. We found potential repurposed drugs namely, Verteporfin, Alatrofloxacin, Metergoline, Rescinnamine, Leuprolide, and Telotristat ethyl with high binding affinity. Furthermore, some of the predicted drugs for the SARS-CoV-2 have already entered the clinical trials as interventional drugs like Argatroban, Metmorfin, Amlodipine, Simvastatin, Isavuconazium and Diosmin. Likewise, some drugs were also predicted through computational approaches by other groups. These findings confirm the predictive power of our computational models. We anticipate these computational methods would assist in antiviral drug discovery against SARS-CoV-2 and other CoVs. In the current scenario of SARS-CoV-2 pandemic, the researchers can directly use the predicted repurposed drug candidates, which would save their money and time in developing the promising therapeutic candidates.

## 4 Material and methods

### 4.1 Datasets

The dataset of the inhibitors of CoVs used in the study has been extracted from our recently published DrugRepV database [32] along with the information of inhibition efficiency, chemical information (SMILES). We used three important CoVs namely SARS, SARS-CoV-2, and MERS in the analysis. Further, we predicted the repurposed drug candidates using MLTs for four categories of viruses *i.e.* overall CoVs, as well as individual SARS-CoV-2, SARS, and MERS. The datasets used in the analysis are available as Supplementary Tables S5-S8.

**Fig. 6:**
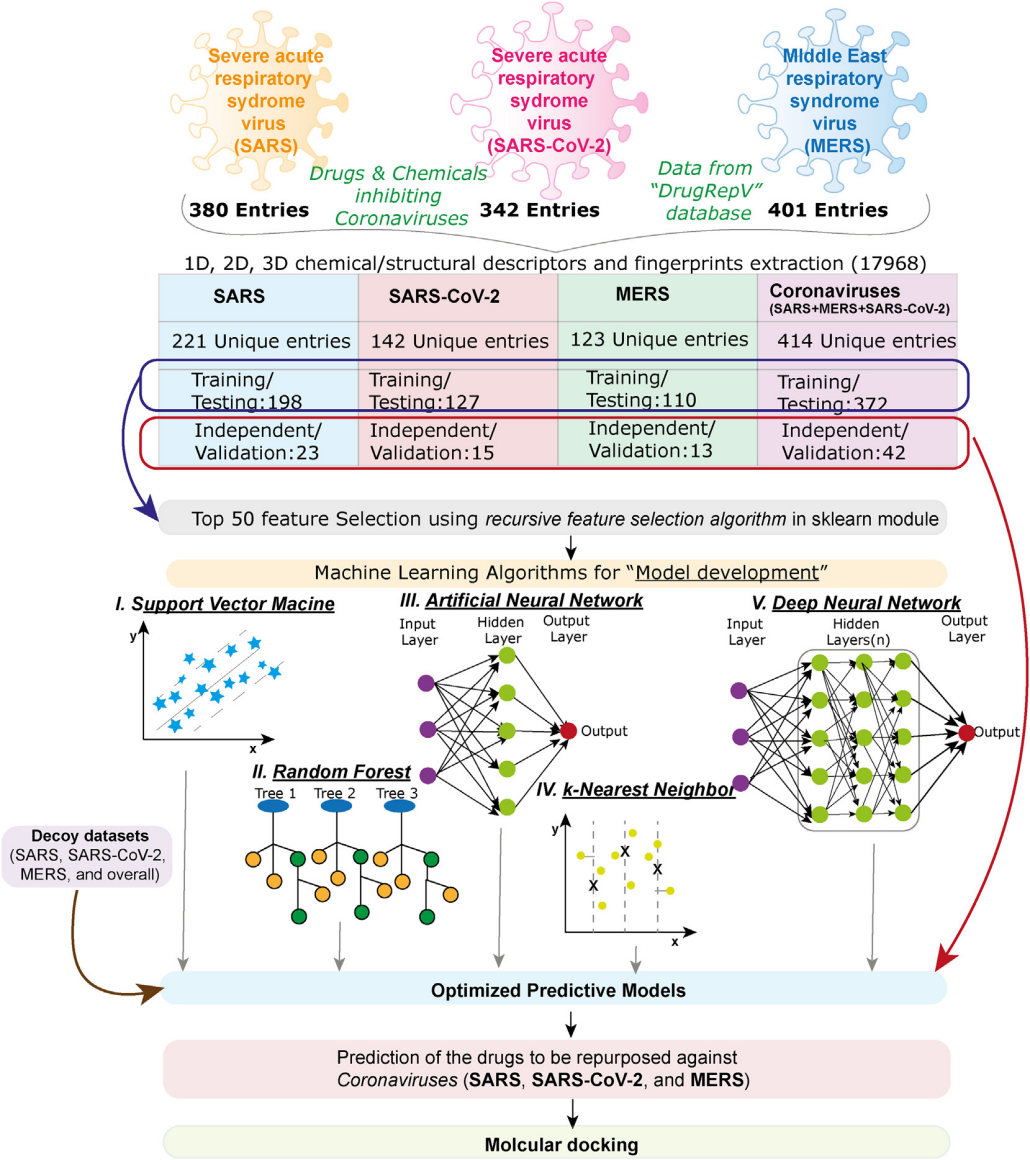
The overall methodology used in the study. The inhibitors of the Coronaviruses (SARS, SARS-CoV-2, and MERS) were extracted from the literature. Splitting of the dataset into the training/testing and independent validation using randomization approach. The descriptors were calculated using PaDel software followed by the selection of relevant features. The prediction model is developed using machine learning algorithms like Support Vector Machine, Random Forest, k-Nearest Neighbor, Artificial Neural Network, and Deep Neural Network.

The overall methodology is described in Fig. 6. The following steps have been used:

1. The SARS, SARS-CoV-2, MERS, and overall CoVs have 380, 342, 401, and 1123 inhibitor entries respectively.
2. Further, quality control involves filtering the entries with IC_50_/EC_50_, SMILES, and unique entries per category.
3. The IC_50_/EC_50_ were converted into the negative logarithm of half-maximal inhibitory concentration (pIC_50_) using the formula (pIC_50_ = –log_10_(IC_50_(M)), where the IC_50_ would be in Molar concentration.
4. After the quality control, we obtained 212, 142, 123, and 414 unique entries for SARS-CoV-2, SARS, MERS, and overall CoVs.
5. The dataset is divided into the training/testing and independent validation datasets using a randomization approach. It resulted in the 221 ^T200+V21^, 142 ^T128+V14^, 123 ^T111+V12^, and 414 ^T373+V41^ entries for SARS, SARS-CoV-2, MERS, and overall CoVs correspondingly.
6. Calculation of the 1D, 2D, 3D, 4D molecular descriptors, and fingerprints was extracted using PaDel software.
7. Feature selection algorithms were performed to get the most relevant features among all four categories.
8. The prediction model is developed using various MLTs like SVM, RF, ANN, KNN, and DNN.

### 4.2 Descriptors extraction

In order to develop the CoVs-specific prediction models, from the anti-corona compounds, we used the PaDEL-Descriptor software [52]. We calculated the 1D, 2D, 3D molecular descriptors, and fingerprints totaling up to 17,968 features. The molecular descriptors are the pieces of information encoded in the molecular structure of a chemical. They are classified according to their dimensionality, *viz*., 1D, 2D, and 3D. The 1D descriptors present the very basic information calculated from the molecular formula like molecular weight. The 2D descriptors like the number of bonds, connectivity indices, etc. describe the signatures calculated from two-dimensional molecular representations, intramolecular hydrogen bonding, etc. The 3D descriptors, as the name suggests, describe the molecular properties related to three-dimensional conformations of the molecule such as solvent accessible surface areas, intramolecular hydrogen bonding, etc. The fingerprints are another way of representing molecules as mathematical objects where binary digits (bits) are used to find and/or differentiate molecular substructures. Together, these descriptors and fingerprints are necessary for establishing a quantitative structure–activity relationship (QSAR) of the chemical compounds under study [53]. These descriptors are very important as used previously in various studies for predicting the inhibitors against various infectious agents [30], [41], [54].

### 4.3 Format conversion

We converted the anticorona chemical compound structures from the simplified molecular-input line-entry system (SMILES) format to the three-dimensional structure-data file (3D-SDF) format using the open-source chemical toolbox Open Babel version 3.0.0 [55]. This format conversion step is necessary for calculating the different descriptors and fingerprints for the curated anti corona chemical compound datasets.

### 4.4 Machine learning algorithms

For the development of the prediction algorithm, we used five different MLTs e.g. SVM, RF, KNN, ANN, and DNN which were called using the SciKit library of Python. While the DNN was run through the Keras Deep Learning Library.

#### 4.4.1 Support Vector Machine

SVM is a supervised MLT used for solving classification and regression-based problems [56]. In the current study, we used SVM for solving the regression problem i.e. Support Vector Regression (SVR). The SVR works on the same principle as for SVM classification, with minor differences. In general, its main focus is minimizing the error, maximizing the margin by individualizing the hyperplane, such that some proportion of the error is being tolerated. It was customized by using the linear and non-linear SVR along with the kernels like Gaussian Radial Basis function and Polynomial.

#### 4.4.2 Random Forest

RF is a supervised learning algorithm that uses an ensemble technique for predicting the classification and regression tasks [57]. It works by forming a forest of multiple decision trees from the training dataset followed by getting the prediction output by taking the mean of the prediction from individual trees for solving a regression task. For getting optimal output from the RF, we used attributes like number of trees (estimators), maximum depth of the trees (max_depth), minimum number of samples required to split an internal node (min_samples_split), minimum number of samples required to be at a leaf node (min_samples_leaf), etc. In the case of the regression problem, it works by taking the mean of the predictions from individual trees.

#### 4.4.3 k-Nearest Neighbor

KNN is a non-parametric MLT and works for both classification and regression problems [58]. It is an instance-based learning or lazy learning method, which depends on the contribution of the local data. It works by spreading the input as the k closest networks in a feature space. For the KNN algorithm, we used different nearest networks i.e. 3, 5, 7, 9, 11, etc.

#### 4.4.4 Artificial Neural network

ANN is a supervised algorithm and consists of nodes and connected units. The collection of connected units and nodes known as artificial neurons, and shows analogy with animal brains [59]. It is an information processing technique, it includes a network of interconnected processing units, which works together to process information and give a meaningful output. For getting the optimized result, we used different activations (*e.g.* tahn, relu), solvers (*e.g.* sgd, adam), and learning rates (*e.g.* constant, invscaling, adative, etc.).

#### 4.4.5 Deep Neural network

DNN is a type of ANN with multiple layers in between input and output layers. It is a feedforward network, where the data moves from input towards the output layers *via* the intermediate layers without moving in the backward direction [60]. It can be used to solve linear as well as complex non-linear relationships. The extra layers help the composition of the features from the lower layers for modeling the very complex data. We used Keras API of the TensorFlow package for solving our regression-based problem. We used a combination of different optimizers (Adam, RMSprop, SGD, Adamax, etc.) and activations (tahn, sigmoid, softmax, etc.) to get the best result. We used 06 intermediate layers with different numbers of neurons in each layer like 256, 128, 64, 32, 16, and 08.

### 4.5 Feature selection

The use of overall extracted 17,968 features in the development of machine learning would lead to various problems like overfitting, curse of dimensionality, etc. In this regard, feature selection would be an important step. We used the Recursive feature elimination (RFE) module of SciKit library in Python. The RFE extracts the features from the training dataset which are more relevant to predict the target variable [61], [62]. In general, it uses two important attributes *i.e.* choice of algorithm and number of the features to be selected. In the current study, we used algorithms within the SVR method in the RFE module.

### 4.6 Performance measures

For regression (quantitative) mode, the correlation between two variables is measured using Pearson’s correlation coefficient (PCC or R). In bioinformatics, the two variables are actual and predicted values. The range of PCC varies from −1 to + 1. If PCC is −1, it indicates that observed and actual values are negatively correlated, 0 shows random prediction, while +1 displays the positive correlation among them. PCC is calculated using formula:

$$\mathrm{PCC}=\frac{n\sum_{n=1}^{n}E_{i}^{\mathrm{act}}E_{i}^{\mathrm{pred}}-\sum_{n=1}^{n}E_{i}^{\mathrm{act}}\sum_{n=1}^{n}E_{i}^{\mathrm{pred}}}{\sqrt{n\sum_{n=1}^{n}{{\left(E_{i}^{\mathrm{act}}\right)}^{2}-\left({\sum_{n=1}^{n}E}_{i}^{\mathrm{act}}\right)}^{2}}-\sqrt{n\sum_{n=1}^{n}{{\left(E_{i}^{\mathrm{pred}}\right)}^{2}-\left({\sum_{n=1}^{n}E}_{i}^{\mathrm{pred}}\right)}^{2}}}$$

where *n,* $E_{i}^{pred}$and $E_{i}^{\mathrm{act}}$ is the size of the test set, predicted and actual efficiencies of CoVs inhibition respectively.

*The coefficient of determination (R^2^)* is the statistical measure of determining the efficiency of a regression line to estimate the real data. The R^2^ varies from 0 to 1, if it is near to 1 means the estimated rate of regression is perfect whereas towards 0 means imperfect estimation.

*Mean Absolute Error (MAE)* is the difference between actual and predicted values.

$$\mathrm{MAE}=\frac{1}{n}\sum_{n=1}^{n}\left|E_{i}^{\mathrm{pred}}-E_{i}^{\mathrm{act}}\right|$$

where, $E_{i}^{\mathrm{pred}}$, $E_{i}^{\mathrm{act}}$ and $\left|E_{i}^{\mathrm{pred}}-E_{i}^{\mathrm{act}}\right|$ are predicted and actual efficiencies of CoVs inhibition and absolute error. The negative values of MAE are preferred for better prediction quality.

*Root Mean Square Error (RMSE)* is the scoring rule to measure the average magnitude of error. Its negative values showed the efficiency of good prediction.

$$\mathrm{RMSE}=\sqrt{\frac{1}{n}\sum_{n=1}^{n}{\left(E_{i}^{\mathrm{pred}}-E_{i}^{\mathrm{act}}\right)}^{2}}$$

where, $E_{i}^{\mathrm{pred}}$ and $E_{i}^{\mathrm{act}}$ are predicted and actual efficiencies of CoVs inhibition.

### 4.7 Applicability domain

The robustness of the predictive developed model was cross-checked by checking the applicability domains [29], [30]. We used William's plot for checking the applicability domain. William's plot was plotted among the leverage and the standardized residuals for training/testing and independent validation datasets. Further, the robustness was also checked by plotting the actual values against the predicted values. The applicability domain was checked for both the training/testing or independent validation dataset. The robust predictive model was shown by the plot if the points of the actual and predictive values localized close to the trend line.

### 4.8 Decoy dataset

Decoy sets were generated for four categories, *i.e.* overall CoVs and individual SARS-CoV-2, SARS, and MERS, using RADER (RApid DEcoy Retriever) software [63]. We have used the default parameters used in the tool, *i.e.* Tanimoto threshold for Active ligand vs. Decoy and Decoy vs. Decoy is 0.75 and 0.50, respectively. For decoy selection, the ZINC database (17,900,742 entries) was selected. Decoys were randomly selected for all the categories using a random number generator program. Using this program, we have developed three random sets for each category of virus. For example, in SARS-CoV-2, each set contains 142 randomly selected decoys. Similarly, random sets developed for SARS (221), MERS (123) and overall (414).

### 4.9 Chemical analysis

Chemical clustering of the SARS, MERS, SARS-CoV-2, and overall unique compounds was done using the ChemMine Tools [64]. We performed the binning clustering using the Tanimoto coefficient (similarity cutoff 0.6). MDS was done at 2D and 3D level using the same similarity threshold. Hierarchical clustering was performed for all the molecules where the heatmaps and circular plots of the heatmaps were constructed for each aforementioned compound group using the ‘distance matrix’ parameter and a ‘single’ linkage method.

### 4.10 Drug repurposing

Repurposing of the drugs against the SARS-CoV-2, SARS, and MERS coronaviruses was done using our developed predicted models. We predicted the repurposed drugs using the best performing SVM models in all three categories. For repurposing the drug categories the “Approved” category of the drugs was downloaded from the DrugBank repository [65]. The descriptors and fingerprints of all the 2468 approved drugs were calculated using the PaDel software. Further, the descriptors of the approved drugs were used to predict the highly efficient drugs against all three categories of viruses.

### 4.11 Molecular docking

The AutoDock tool (ADT) was used to customize the ligand and Protein [66]. Further, their molecular structure was saved in PDBQT file format. The AutoDock Vina (*v*1.1.2) [67] was used at default parameter to perform the docking between the SARS-CoV-2 S-protein complex with ACE-2 receptor (PDB: 6lzg) [68] and predicted inhibitors. The grid box was generated at center_x = -26.908, center_y = 18.289, center_z = -13.883, spacing 0.375- Å, size_x = 40, size_y = 40, size_z = 40. Subsequently, protein and ligand molecules were docked to generate the 09 best docking poses. To find the minimum binding affinity the exhaustiveness parameter was set to10. The ligand and protein molecules interacting residues were analysed using Pymol [44] and Discovery Studio Visualizer [69].

## 5 Code availability

The Python code used in study is provided on GitHub (https://github.com/manojk-imtech/antiCorona).

## 6 Authors’ contribution

MK conceived the idea and helped in the interpretation, analysis, and overall supervision. AR and AT performed data collection and curation. AR developed the predictive AI and MLT pipelines. AT, AM, SK, AMR, SG implemented model development. AR, AT, AM, SK, MK did decoy set analysis. AR, AT, AM did chemical analysis. AR, AT, and HJ involved in data visualization. AMR performed the molecular docking. AR, AT, AM, SK, AMR, SG, and MK wrote the manuscript.

## Funding

This work was supported by the grants from the CSIR-Institute of Microbial Technology, Council of Scientific and Industrial Research (CSIR) (OLP0501, OLP0143 and STS0038).

## CRediT authorship contribution statement

**Akanksha Rajput:** Methodology, Data curation, Software, Investigation, Validation, Formal analysis, Visualization, Writing - original draft, Writing - review & editing. **Anamika Thakur:** Methodology, Data curation, Validation, Formal analysis, Visualization, Writing - original draft. **Adhip Mukhopadhyay:** Validation, Formal analysis, Writing - original draft. **Sakshi Kamboj:** Validation, Formal analysis, Writing - original draft, Writing - review & editing. **Amber Rastogi:** Validation, Formal analysis, Writing - original draft. **Sakshi Gautam:** Validation, Writing - original draft, Writing - review & editing. **Harvinder Jassal:** Visualization. **Manoj Kumar:** Conceptualization, Supervision, Formal analysis, Funding acquisition, Project administration, Writing - original draft, Writing - original draft, Writing - review & editing.

## Declaration of Competing Interest

The authors declare that they have no known competing financial interests or personal relationships that could have appeared to influence the work reported in this paper.
