# Supplementary material for: Prediction of repurposed drugs for Coronaviruses using artificial intelligence and machine learning
Source: Comput Struct Biotechnol J. 2021 May 24;19:3133–48. doi: 10.1016/j.csbj.2021.05.037 (PMC8141697; doi:10.1016/j.csbj.2021.05.037)
Supplement: Supplementary data 1 [file mmc1.pdf]

Prediction of repurposed drugs for Coronaviruses using artificial intelligence and machine learning

Akanksha Rajput[1], Anamika Thakur[1,2], Adhip Mukhopadhyay[1,2], Sakshi[1,2], Amber Rastogi[1,2], Sakshi Gautam[1,2], Harvinder Jassal[1] and Manoj Kumar[1,2 *]

[1]Virology Unit and Bioinformatics Centre, Institute of Microbial Technology, Council of Scientific and Industrial Research (CSIR), Sector 39-A, Chandigarh-160036, India
[2]Academy of Scientific and Innovative Research (AcSIR), Ghaziabad-201002, India
* To whom correspondence should be addressed. Tel: +91 172 6665158; Fax: +91 172 2690585; Email: manojk@imtech.res.in

Supplementary information

Supplementary Figure S1. *The chemical analysis a) The 3-dimensional multiscaling plot among the SARS inhibitors. b) The 3-dimensional multiscaling plot among the MERS inhibitors.*

Supplementary Figure S2. *The chemical network of the predicted repurposed drug candidates which are common between MERS and SARS viruses*

Supplementary Figure S3. *The chemical network of the predicted repurposed drug candidates which are common between MERS and SARS-CoV-2 viruses*

Supplementary Figure S4. *The chemical network of the predicted repurposed drug candidates which are common between SARS-CoV-2 and SARS viruses*

Supplementary Figure S5. *The network of the predicted repurposed drug candidates in SARS-CoV-2. The predicted drugs are categorized in five groups like clinical trials (intervational), Clinical trials (observational), Clinical trials (non relevant), experimental, and computational*

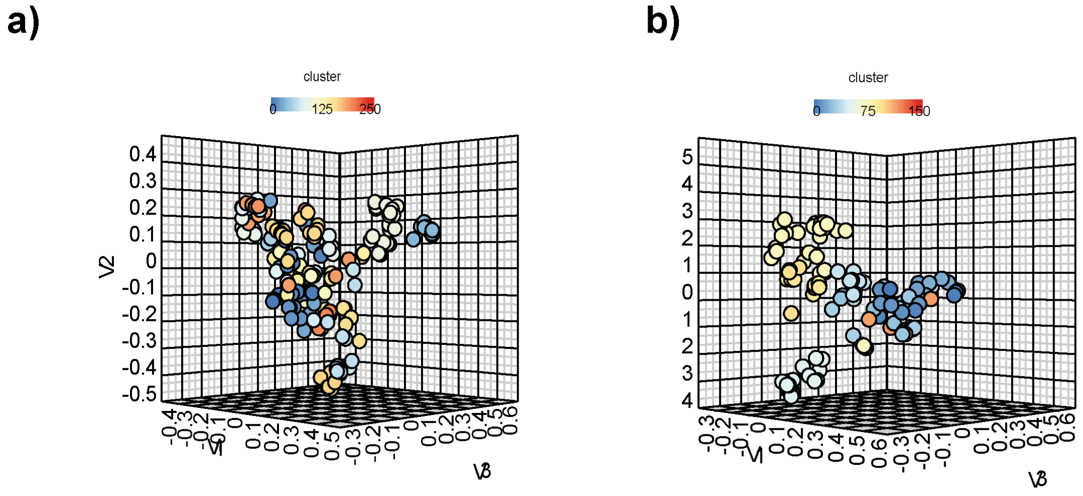

Supplementary Figure S1. *The chemical analysis of the Severe Acute Respiratory Syndrome (SARS) inhibitors a) The 3-dimensional multiscaling plot among the SARS inhibitors. b) The 3-dimensional multiscaling plot among the MERS inhibitors.*

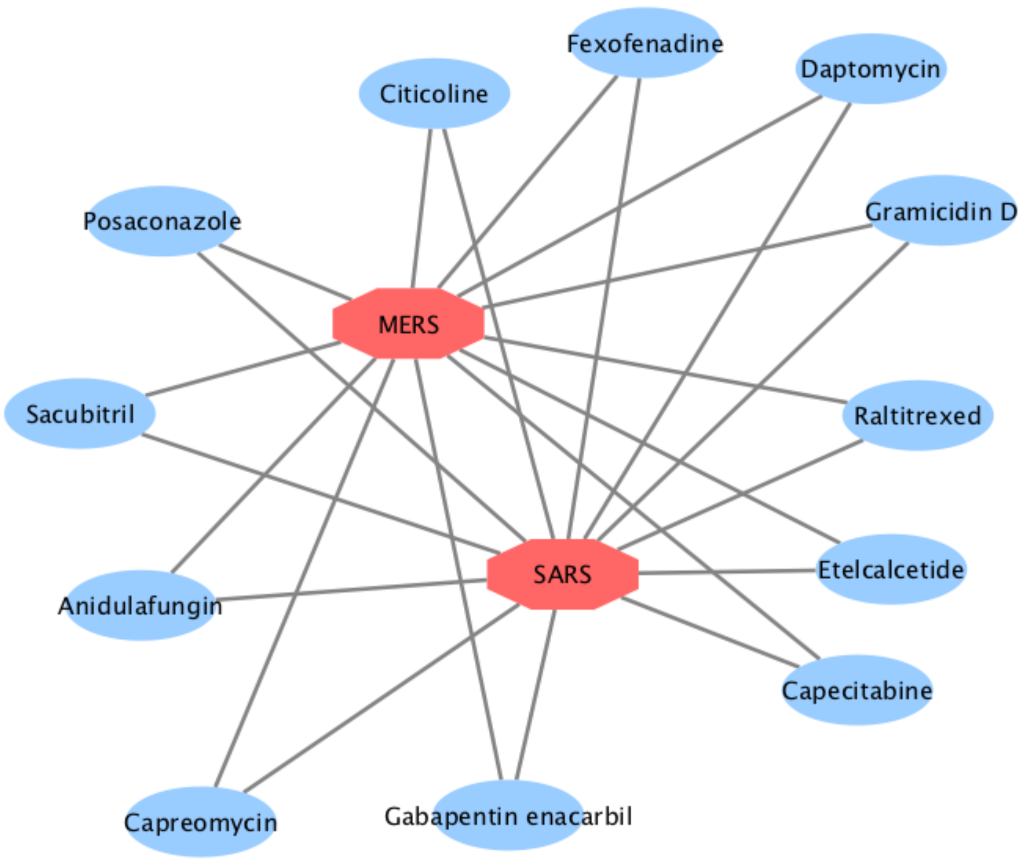

Supplementary Figure S2. *The chemical network of the predicted repurposed drug candidates which are common between MERS and SARS viruses*

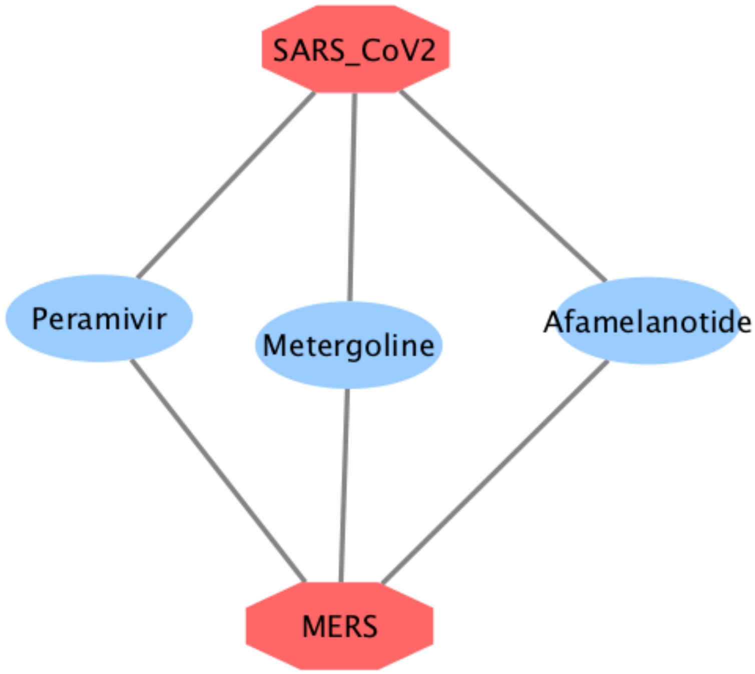

Supplementary Figure S3. *The chemical network of the predicted repurposed drug candidates which are common between SARS-CoV-2 and MERS viruses*

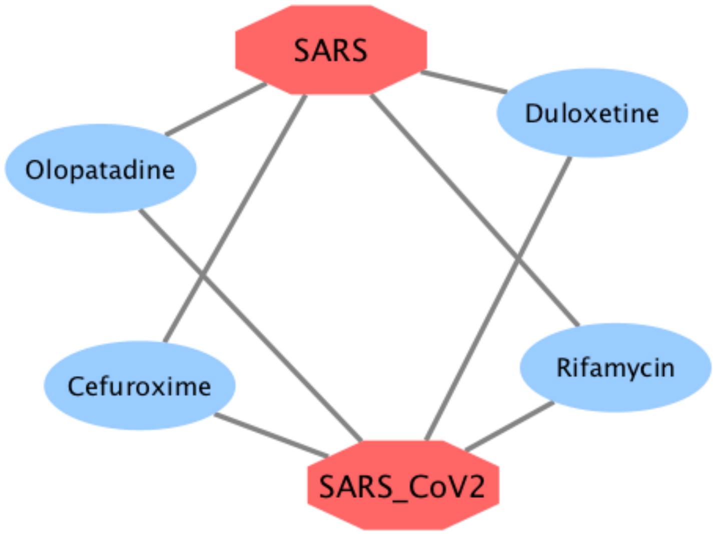

Supplementary Figure S4. *The chemical network of the predicted repurposed drug candidates which are common between SARS-CoV-2 and SARS viruses*

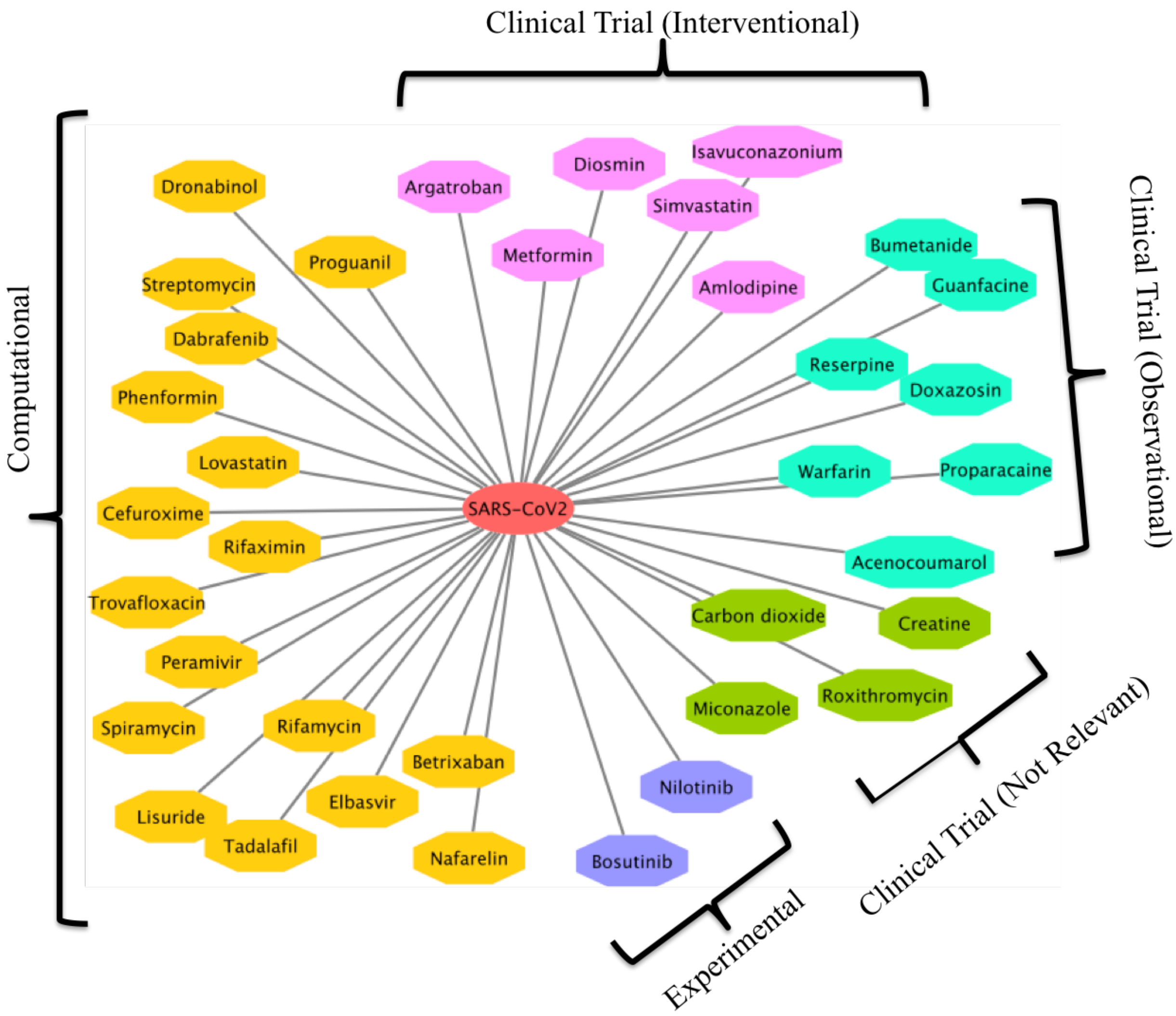

Supplementary Figure S5. *The network of the predicted repurposed drug candidates in SARS-CoV-2. The predicted drugs are categorized in five groups like clinical trials (intervational), Clinical trials (observational), Clinical trials (non relevant), experimental, and computational*
